# Supplementary material for: The Systems Biology Research Tool: evolvable open-source software
Source: BMC Syst Biol. 2008 Jun 29;2:55. doi: 10.1186/1752-0509-2-55 (PMC2446383; doi:10.1186/1752-0509-2-55)
Supplement: Additional file 1 — SBRT Archive. An archive of the current version of the Systems Biology Research Tool. [file 1752-0509-2-55-S1.zip › sbrt-1.4.0/doc/developers_guide/api/sbrt/kernel/math/algebra/MatrixMultiplier.html]

MatrixMultiplier


|  |  |  |
| --- | --- | --- |
| **Overview** \| **Package** \| **Class** \| **Use** \| **Tree** \| **Deprecated** \| **Index** \| **Help** | |  |
| **PREV CLASS**   **NEXT CLASS** | **FRAMES**    **NO FRAMES**     **All Classes** |  |
| SUMMARY: NESTED \| FIELD \| CONSTR \| METHOD | DETAIL: FIELD \| CONSTR \| METHOD |  |


---


## sbrt.kernel.math.algebra Class MatrixMultiplier

```
java.lang.Object
  sbrt.kernel.math.algebra.MatrixMultiplier
```

---

``` public class MatrixMultiplier extends java.lang.Object ```

This class is used to multiply matrices.

**Author:**
:   This class was written and documented by Jeremiah Wright
    while in the Wagner lab.

---

**Constructor Summary**

`MatrixMultiplier()`


**Method Summary**

`<R,C,T> void`

`multiply(SparseDoubleMatrix<R,C> matrixA,
SparseDoubleMatrix<C,T> matrixB,
DoubleMatrixBuilder<R,T> product)`
  
          Fills `product` with `matrixA` \* `matrixB`.

**Methods inherited from class java.lang.Object**

`clone, equals, finalize, getClass, hashCode, notify, notifyAll, toString, wait, wait, wait`

**Constructor Detail**

### MatrixMultiplier

```
public MatrixMultiplier()
```


**Method Detail**

### multiply

```
public <R,C,T> void multiply(SparseDoubleMatrix<R,C> matrixA,
                             SparseDoubleMatrix<C,T> matrixB,
                             DoubleMatrixBuilder<R,T> product)
```

:   Fills `product` with `matrixA` \* `matrixB`.

    :   **Type Parameters:**: `R` - the row type of `matrixA`.: `C` - the column type of `matrixA` and row type of `matrixB`.: `T` - the column type of `matrixB`. **Parameters:**: `matrixA` - a matrix.: `matrixB` - a matrix.: `product` - an empty matrix.


---


|  |  |  |
| --- | --- | --- |
| **Overview** \| **Package** \| **Class** \| **Use** \| **Tree** \| **Deprecated** \| **Index** \| **Help** | |  |
| **PREV CLASS**   **NEXT CLASS** | **FRAMES**    **NO FRAMES**     **All Classes** |  |
| SUMMARY: NESTED \| FIELD \| CONSTR \| METHOD | DETAIL: FIELD \| CONSTR \| METHOD |  |


---
